# Controlled Hemorrhage Sensitizes Angiotensin II-Elicited Hypertension through Activation of the Brain Renin-Angiotensin System Independently of Endoplasmic Reticulum Stress

**DOI:** 10.1155/2022/6371048

**Published:** 2022-01-13

**Authors:** Guo-Biao Wu, Hui-Bo Du, Jia-Yi Zhai, Si Sun, Jun-Ling Cui, Yang Zhang, Zhen-Ao Zhao, Jian-Liang Wu, Alan Kim Johnson, Baojian Xue, Zi-Gang Zhao, Geng-Shen Zhang

**Affiliations:** ^1^Departments of Neurosurgery and Medical Equipment, Second Hospital, Hebei Medical University, Shijiazhuang City, Hebei, China; ^2^Institute of Microcirculation, Hebei Key Laboratory of Critical Disease Mechanism and Intervention, Hebei North University, Zhangjiakou City, Hebei, China; ^3^Department of Psychological and Brain Sciences, University of Iowa, Iowa City, IA, USA

## Abstract

Hemorrhagic shock is associated with activation of renin-angiotensin system (RAS) and endoplasmic reticulum stress (ERS). Previous studies demonstrated that central RAS activation produced by various challenges sensitizes angiotensin (Ang) II-elicited hypertension and that ERS contributes to the development of neurogenic hypertension. The present study investigated whether controlled hemorrhage could sensitize Ang II-elicited hypertension and whether the brain RAS and ERS mediate this sensitization. Results showed that hemorrhaged (HEM) rats had a significantly enhanced hypertensive response to a slow-pressor infusion of Ang II when compared to sham HEM rats. Treatment with either angiotensin-converting enzyme (ACE) 1 inhibitor, captopril, or ACE2 activator, diminazene, abolished the HEM-induced sensitization of hypertension. Treatment with the ERS agonist, tunicamycin, in sham HEM rats also sensitized Ang II-elicited hypertension. However, blockade of ERS with 4-phenylbutyric acid in HEM rats did not alter HEM-elicited sensitization of hypertension. Either HEM or ERS activation produced a greater reduction in BP after ganglionic blockade, upregulated mRNA and protein expression of ACE1 in the hypothalamic paraventricular nucleus (PVN), and elevated plasma levels of Ang II but reduced mRNA expression of the Ang-(1-7) receptor, Mas-R, and did not alter plasma levels of Ang-(1-7). Treatment with captopril or diminazene, but not phenylbutyric acid, reversed these changes. No treatments had effects on PVN protein expression of the ERS marker glucose-regulated protein 78. The results indicate that controlled hemorrhage sensitizes Ang II-elicited hypertension by augmenting RAS prohypertensive actions and reducing RAS antihypertensive effects in the brain, which is independent of ERS mechanism.

## 1. Introduction

Hemorrhagic shock (HS) is characterized by a rapid and significant loss of blood that leads to hemodynamic instability, decreased tissue perfusion, organ injury, and even death [1]. The current treatments for HS include timely hemostasis, volume replacement, and whole blood or blood component therapy [2–4]. There have been progress in understanding hemorrhage pathophysiology and improvement in the treatment to increase survival [5]. However, the long-term consequences of HS and the development of chronic disorders, such as cardiovascular diseases including hypertension, and interventions to reduce such pathologies in survivors of HS have not been reported.

HS is associated with activation of the sympathetic nervous system (SNS), renin-angiotensin system (RAS), and arginine vasopressin (AVP). These three systems are mobilized to restore homeostasis following acute HS, and elimination of any of these pressor systems attenuates the compensatory response to acute hypovolemic hypotension [6–10]. For example, central blockade of angiotensin II type 1 receptor (AT1-R) produced a markedly greater fall in blood pressure (BP), a reduced tachycardia, and impaired vasopressin release during and after hemorrhage, suggesting that brain angiotensin (Ang) II acting through AT1-R plays an important physiological role in mediating rapid cardiovascular regulation in response to hemorrhage [7, 11].

Recent studies have demonstrated that hemorrhage leads to endoplasmic reticulum stress (ERS) that is initiated by the elevated inflammation and overactivation of the RAS following hemorrhage [12–14]. All these responses to hemorrhage depend on the integrity of several brain regions controlling SNS tone and BP regulation including periventricular tissue surrounding the anteroventral third ventricle (AV3V) region and the hypothalamic paraventricular nucleus (PVN) [15–19].

It is well established that interactions between the RAS and proinflammatory cytokines (PICs, e.g., tumor necrosis factor alpha (TNF-*α*), interleukin- (IL-) 1*β*, and IL-6) play a critical role in the development of neurogenic hypertension via their actions in various brain cardiovascular nuclei such as the subfornical organ (SFO), PVN, and rostral ventrolateral medulla (RVLM) [20–23]. Many types of hypertension increase brain Ang II formation and upregulate expression of brain AT1-R and angiotensin-converting enzyme (ACE) 1 [24, 25]. Another form of ACE, ACE2, is a component of the antihypertensive arm of the RAS that has properties opposite to those of the classic prohypertensive arm of the RAS. ACE2 acts to convert Ang II into Ang-(1–7) [26]. Reduced ACE2 expression and/or enzyme activity have been found in various brain regions in hypertension models [27, 28]. Overexpression of ACE2 in the brain blunts the development of hypertension in several animal models, including Ang II-induced and DOCA/salt-induced hypertension [27, 29, 30]. Recently, ERS has been implicated in the development and maintenance of neurogenic hypertension. Activation of ERS in the SFO and the RVLM mediates Ang II-induced hypertension and increased BP in spontaneously hypertensive rats (SHRs) [31, 32]. Therefore, both hemorrhage and hypertension can activate common mediators and act on the same cardiovascular nuclei in the physiological and pathophysiological process.

Many physiological stressors have been shown to induce hypertensive response sensitization (HTRS). Physiological stressors also induce upregulation of several prohypertensive components of the brain RAS and PICs. Such changes in the central nervous system (CNS) have been found in key components of the central neural network controlling sympathetic drive. These areas include the PVN and components of the lamina terminalis (LT), namely, the SFO, the median preoptic nucleus (MnPO), and the organum vasculosum (OVLT). In earlier preliminary studies, we found that hypotensive hemorrhage induced HTRS [33]. Given that hemorrhage activates the brain RAS, inflammation, and ERS [12–14] and activation of the prohypertensive arm of the RAS and inflammation in the brain are involved in the sensitization of hypertension [21, 22], we hypothesized that controlled hypotensive hemorrhage inducing HTRS is mediated by the RAS and ERS. To test the hypothesis, we first investigated the sensitizing effect of controlled hemorrhage or activation of ERS on HTRS elicited by a slow-pressor Ang II challenge. Because blockade of ACE1 or ERS improves the hemodynamic and metabolic status and attenuates acute organ injury in HS animals [34–36], we also determined whether systemic treatment with captopril (Cap, ACE1 inhibitor), diminazene aceturate (DIZE, ACE2 activator), or 4-phenylbutyric acid (4-PBA, ERS antagonist) would have long-term beneficial effects by blocking hemorrhage-induced sensitization. We also tested whether either activation or inhibition of activity in components of the brain RAS, PICs, and ERS altered the effect of hemorrhage on the induction of HTRS.

## 2. Materials and Methods

### 2.1. Animals

All procedures were reviewed and approved by the Hebei Medical University and Hebei North University Institutional Animal Care and Use Committee conforming to US National Institutes of Health guidelines. All experiments were performed in the Hebei North University.

Ten-week-old male Wistar rats were purchased from Sibeifu Biotechnology Co., Ltd (Beijing, China), housed in standard plastic microisolator cages, and maintained in a temperature- (23 ± 2°C) and light- (12-h light/dark cycle) controlled animal facility. The rats had unlimited access to standard rat chow and water. A total of ninety-seven male rats were used for the experiments.

### 2.2. Controlled Hemorrhage Paradigm

Rat transmitters (HD-S10, DSI®, St. Paul, MN) were used to directly measure BP and heart rate (HR). After baseline BP and HR recordings were made, rats were hemorrhaged (HEM) via the jugular vein on two occasions separated by 1 day. The blood was collected in heparin. Then, the blood was centrifuged, and the cells were resuspended in saline for reinfusion. Hemorrhage proceeded at the rate of 1.5 ml/min (a total volume usually 5 ml/rat) until BP was lowered to ~40 mmHg, and then, BP was maintained at that level for 30 min after which time the rats were reinfused. Rats in the sham HEM (S-HEM) group underwent the same surgery and procedure, but there was no blood withdrawal. After one week, the rats were administrated a slow-pressor dose of Ang II (120 ng/kg/min, sc) for 2 weeks to test for HTRS.

*Experiment 1*. BP and HR were recorded by telemetry for 5 days at baseline and then for the subsequent 24 consecutive days. Over this time, rats were first subjected to the two S-HEM or HEM procedures and then 8 days later by implantation of an osmotic minipump (model 2002, ALZET) that delivered Ang II (120 ng/kg/min, sc) for the next two weeks. Some groups of S-HEM and HEM rats were treated with RAS or ERS agonists or antagonists beginning immediately after the first S-HEM/HEM procedures and continuing until immediately before beginning testing for HTRS elicited by the Ang II infusion. Therefore, the experiments involved 6 groups (*n* = 5-7 rats/group): (1) S-HEM+Ang II; (2) HEM+Ang II; (3) HEM/Cap+Ang II (ACE1 inhibitor, 40 mg/kg/day, ip); (4) HEM/DIZE+Ang II (ACE2 activator, 7.5 mg/kg/day, ip); (5) HEM/4-PBA+Ang II (ERS antagonist, 100 mg/kg, ip); or (6) S-HEM/tunicamycin+Ang II (TM, ERS agonist, 10 *μ*g/kg, ip). The doses of Cap, DIZE, 4-PBA, and TM were chosen based on published studies, and they were injected ip daily [37–40].

*Experiment 2*. Additional studies were performed to assess the effects of the S-HEM and HEM on plasma levels of Ang II and Ang-(1-7) as well as gene and protein expression of RAS and PIC components and ERS marker in the PVN. Trunk blood and brains of S-HEM rats, HEM rats, and HEM rats treated with Cap, DIZE, 4-PBS, or TM (*n* = 10 per group) were collected on day 8 after the last HEM, corresponding to the time at which Ang II infusion was initiated in Experiment 1.

### 2.3. Telemetry Transmitter and Osmotic Pump Implantation

The rats were anesthetized with a ketamine-xylazine mixture (90% ketamine and 10% xylazine), and the femoral artery was accessed with a ventral incision. The right femoral artery was isolated, and the catheter of a telemetry probe was inserted into the vessel. Through the same ventral incision, a pocket along the right flank was formed. The body of the telemetry transmitter (HD-S10, DSI®, St. Paul, MN) was slipped into the pocket and secured with tissue adhesive. The ventral incision was then closed with suture. Beginning seven days after surgery, BP and HR data collection was initiated.

In a separate procedure under isoflurane anesthesia (0.5-5% inhalation), osmotic pumps (model 2002, ALZET®) containing Ang II (120 ng/kg/min, Sigma) were implanted subcutaneously in the back of rats.

### 2.4. Evaluation of BP Responses to Autonomic Blockade

BP was also measured in the presence of the ganglionic blocker hexamethonium (Hex, 30 mg/kg, ip). Ganglionic blockade was repeated two times in each animal once during baseline and once after 14 days of Ang II infusion. On the day of ganglionic blockade experiments, rats were allowed to stabilize for at least 60 min, after which time BP was recorded for 20 min before and after Hex injection.

### 2.5. Real-Time PCR Analysis

In Experiment 2, all rats were decapitated, and the brains were quickly removed and put in iced saline for 1 minute. Then, the brains were cut into coronal sections of approximately 200 *μ*m thickness, and PVN tissues were punched with a 15-gauge needle stub (inner diameter: 1.5 mm). Some immediately surrounding tissue was usually included in the punch biopsies. Both sides of PVN were analyzed for mRNA expression as a whole. Total RNA was isolated from the PVN using the Trizol method (Invitrogen) and treated with DNase I (Invitrogen, Carlsbad, CA, USA) to remove any genomic DNA contamination. RNA integrity was checked by gel electrophoresis. Total RNA was reverse transcribed following the manufacturer's instructions (Applied Biosystems, Foster City, CA, USA). Real-time PCR was conducted using 200-300 ng of cDNA and 500 nM of each primer in a 20 *μ*l reaction with iQ SYBR Green Supermix (Bio-Rad, Hercules, CA, USA). Amplification cycles were conducted at 95°C for 3 min, followed by 40 cycles of 95°C for 15 s and annealing/extension at 60°C for 30 s. Reactions were performed in duplicate and analyzed using a C1000 thermocycler system (Bio-Rad). Messenger RNA levels for RAS components (renin, ACE1, AT1-R, ACE2, Ang-(1-7) receptor Mas-R), PICs (TNF-*α*, IL-1*β*, and IL-6), ERS marker (glucose-regulated protein 78 (GRP 78)), and GAPDH were analyzed with SYBR Green real-time RT-PCR. The sequences for the primers are summarized in Table 1. Real-time RT-PCR was performed with the ABI prism 7300 Sequence Detection System (Applied Biosystems, Carlsbad, CA). The values were corrected by GAPDH, and the final concentration of mRNA was calculated using the formula *x* = 2^−ΔΔCt^, where *x* is the fold difference relative to control.

### 2.6. Western Blot Analysis

PVN tissues were homogenized in lysis buffer, and the protein concentration in the supernatant was measured with the BCA protein assay Kit (Pierce, Rockford, IL, USA). Equivalent amounts of protein were separated on 4-15% SDS-polyacrylamide gels and transferred to polyvinylidene difluoride membranes (Millipore Corporation, Bedford, MA, USA). The membranes were blocked in 5% nonfat dry milk for 1 h and then incubated with primary antibodies to ACE1 (1 : 100, ab11734, Abcam, Cambridge, MA, USA), ACE2 receptor (1 : 500, ab108252, Abcam), GRP78 (1 : 400, ab21685, Abcam), and *β*-actin (1 : 5000, 66009-1-1 g, Proteintech, Rosemont, IL, USA) overnight at 4°C. After three washing, the membranes were incubated with horseradish peroxidase-conjugated secondary antibodies (Applygen, Beijing, China) for 1 h at room temperature. The signal was visualized using an enhanced chemiluminescence (ECL) detection system (ImageQuant LAS 4000, GE Co., Boston, MA, USA), and densities of the immunobands were quantitated using Quantity One software (V4.6.2, Bio-Rad Co., Boston, MA, USA). All data were corrected by *β*-actin.

### 2.7. Blood Plasma Analysis

In Experiment 2, when the rats were decapitated, trunk blood was collected in a sodium heparin tube (BD vacutainer) and centrifuged. The plasma was used for biochemical assays. Plasma levels of Ang II (Cat#, E-EL-R1430c, Elabscience Biotechnology, Wuhan, China) and Ang-(1-7) (Cat#, E-EL-R1138c, Elabscience Biotechnology, Wuhan, China) were measured with commercial ELISA kits according to the manufacturers' instructions.

### 2.8. Statistical Analysis

Mean arterial pressure (MAP) and HR obtained from the telemetry recordings are presented as mean daily values averaged from daytime and nighttime measurements. Differences for MAP and HR were calculated for each animal based on the mean of a 5-day baseline subtracted from the mean of the final 5 days of Ang II treatment. For experiments on the effect of acute Hex injection, differences for BP were calculated for each animal based on the baseline subtracted from the BP after ip injection of Hex. Two-way ANOVAs for the experimental groups were then conducted on the means of the calculated differences for MAP and HR. After establishing a significant ANOVA, post hoc analyses were performed with Tukey multiple comparison tests between pairs of mean changes (GraphPad Prism 8.0). One-way ANOVAs and post hoc Tukey analyses were used to test for the differences in plasma levels and mRNA and protein expression of the RAS and PIC components and ERS marker in the blood and PVN, respectively. All data are expressed as means ± SEM. Statistical significance was set at *p* < 0.05.

## 3. Results

### 3.1. HEM Sensitizes Systemic Ang II-Induced Hypertension and the Effects of Treatment with Cap or DIZE on HEM-Induced Sensitization of Hypertension

**Figure 1:**
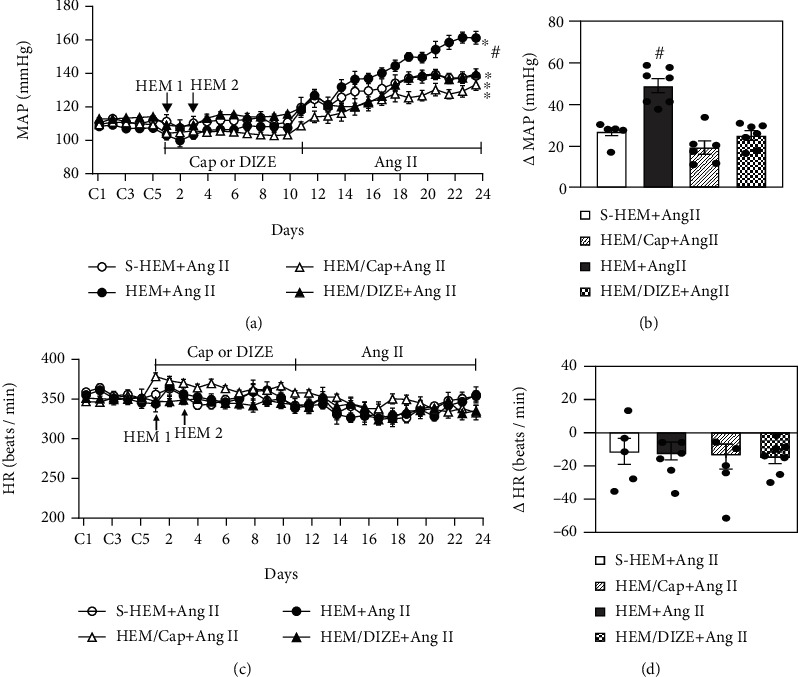
Hypertensive response (a, b) and heart rate (HR) changes (c, d) induced by angiotensin (Ang) II in sham hemorrhage (S-HEM), HEM, and HEM plus treatment with either captopril (Cap) or diminazene aceturate (DIZE) rats. The enhanced hypertensive response in HEM rats was attenuated by either Cap or DIZE treatment. Baseline recordings are denoted by C's (*n* = 5-7/group; ^∗^*p* < 0.05 vs. baseline; ^#^*p* < 0.05 vs. S-HEM or HEM rats plus Cap or DIZE treatment).

During infusion with the slow-pressor dose of ANG II, the HEM rats showed a significantly enhanced hypertensive response (Δ49.2 ± 3.3 mmHg) compared with S-HEM rats (Δ26.9 ± 1.9 mmHg, *p* < 0.05, Figures 1(a) and 1(b)).

Treatment with Cap beginning at the end of the first HEM procedure and continuing until starting the infusion of the slow-pressor dose of Ang II produced a slight decrease in MAP (104.3 ± 1.2 mmHg, *p* < 0.05) and increase in HR (366.8 ± 4.3 beats/min, *p* < 0.05) when compared to baseline measures (MAP, 109.9 ± 1.2 mmHg; HR, 349.7 ± 3.1 beats/min). Treatment with DIZE had no effects on basal MAP or HR. However, treatment with either the ACE1 inhibitor Cap or the ACE2 activator DIZE abolished the HEM-induced sensitization of hypertension in the HEM rats (Cap, Δ19.4 ± 3.1 mmHg; DIZE, Δ25.1 ± 2.2 mmHg, *p* < 0.05, Figures 1(a) and 1(b)) (two-way ANOVA for changes in MAP, *F*(3, 15) = 19.72, *p* < 0.001).

Chronic Ang II infusion did not produce significant changes in HR in any of the groups (two-way ANOVA for changes in HR, *F*(3, 15) = 0.09599, *p* = 0.9611) (Figures 1(c) and 1(d)).

### 3.2. The Effects of Treatment with ERS Antagonist or Agonist on HEM-Induced Sensitization of Hypertension

**Figure 2:**
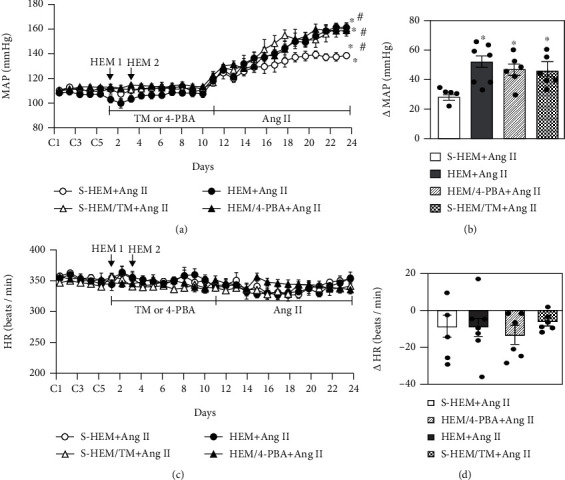
Pressor effect (a, b) and heart rate (HR) changes (c, d) induced by angiotensin (Ang) II in sham hemorrhage (S-HEM), HEM, and HEM plus treatment with either 4-phenylbutyric acid (4-PBA) or tunicamycin (TM) rats. Activation of endoplasmic reticulum stress (ERS) by TM resulted in sensitization of Ang II-induced hypertension. However, the sensitized hypertensive response in HEM rats was not attenuated by blockade of ER stress by 4-PBA treatment. Baseline recordings are denoted by C's (*n* = 5-7/group; ^∗^*p* < 0.05 vs. baseline; ^#^*p* < 0.05 vs. S-HEM rats).

Treatment with either the ERS antagonist 4-PBA or agonist TM from the end of the first S-HEM or HEM treatment to the beginning of infusion of the slow-pressor dose of Ang II produced no effects on baseline MAP and HR. Like the sensitizing effect of HEM on Ang II-induced hypertension, treatment with TM in S-HEM rats also sensitized the hypertensive response to systemic Ang II (Δ45.6 ± 4.4 mmHg) when compared to that in S-HEM alone (Δ26.9 ± 1.9 mmHg, *p* < 0.05, Figures 2(a) and 2(b)). However, blockade of ERS by treatment with 4-PBA did not alter the HEM-induced sensitization of hypertension in the HEM rats (Δ49.2 ± 3.3 mmHg, *p* > 0.05, Figures 2(a) and 2(b)) (two-way ANOVA for changes in MAP, *F*(3, 14) = 5.736, *p* = 0.0089).

Chronic Ang II infusion produced comparable changes in HR in all groups (two-way ANOVA for changes in HR *F*(3, 14) = 0.5714, *p* = 0.643) (Figures 2(c) and 2(d)).

### 3.3. Effects of Autonomic Blockade on BP

**Figure 3:**
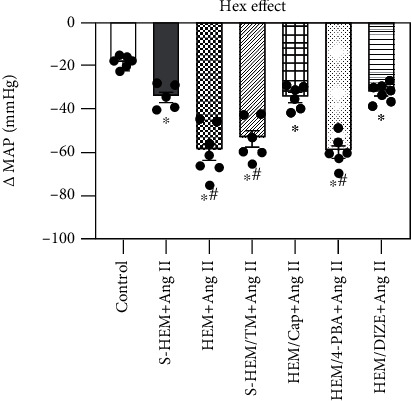
Mean arterial pressure (MAP) in response to ganglionic blockade with hexamethonium (Hex) before hemorrhage (HEM) (control) and on day 14 of angiotensin (Ang) II infusion in all groups (^∗^*p* < 0.05 vs. control, ^#^*p* < 0.05 vs. S-HEM or HEM rats plus captopril (Cap) or diminazene aceturate (DIZE) treatment).

Figure 3 shows decreases in BP with acute ganglionic blockade in all groups. The average reduction in the BP response to Hex injection before S-HEM or HEM was −18.8 ± 0.9 mmHg. Following 14 days of Ang II infusion, acute Hex injection resulted in a significant reduction in BP in S-HEM rats (−34.7 ± 2.0 mmHg, *p* < 0.05). However, the reductions in BP after Hex injection were further augmented in HEM rats (−59.6 ± 4.2 mmHg, *p* < 0.05) or S-HEM rats treated with ER stress agonist (−53.7 ± 3.6 mmHg, *p* < 0.05). Treatments with ACE1 inhibitor (−34.8 ± 2.0, *p* < 0.05) or ACE2 activator (−32.5 ± 1.5 mmHg, *p* < 0.05) in HEM rats significantly attenuated the Hex-induced reduction in BP, but treatment with the ERS blocker in HEM rats did not alter it (−59.7 ± 2.6 mmHg, *p* > 0.05) (two-way ANOVA for changes in BP, *F*(6, 30) = 27.66, *p* < 0.0001).

### 3.4. Changes in Plasma Levels of Ang II and Ang-(1-7)

**Figure 4:**
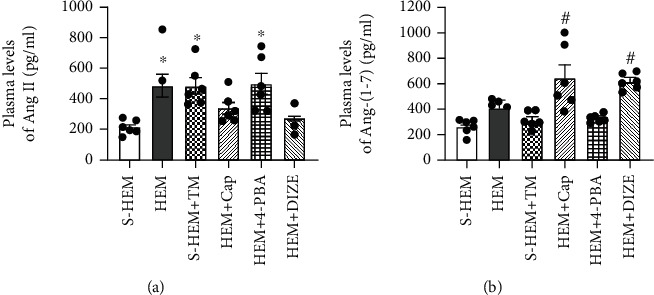
Comparisons of plasma angiotensin (Ang) II (a) and Ang-(1-7) (b) in sham hemorrhage (S-HEM), HEM, and HEM plus treatment with captopril (Cap), diminazene aceturate (DIZE), 4-phenylbutyric acid (4-PBA), or tunicamycin (TM) rats before Ang II infusion (^∗^*p* < 0.05 vs. S-HEM or HEM rats plus Cap or DIZE treatment; ^#^*p* < 0.05 vs. S-HEM, HEM, or HEM rats plus 4-PBA or TM treatment).

Plasma levels of Ang II were markedly increased in HEM rats when compared to those in S-HEM rats (*p* < 0.05). However, in rats treated with either the ACE1 inhibitor or the ACE2 activator, HEM did not induce an increase in plasma Ang II levels when compared to S-HEM rats (*p* > 0.05). Like the HEM, ERS agonist TM also produced a significant increase in Ang II levels in S-HEM rats (*p* < 0.05), but the ERS antagonist 4-PBA had no inhibitory effect on HEM-induced increase in Ang II levels and Ang II levels remained high (*p* > 0.05) (one-way ANOVA for changes in Ang II levels, *F*(5, 30) = 5.724, *p* < 0.0008) (Figure 4(a)).

Either HEM or ERS activation or inhibition had no effects on plasma levels of Ang-(1-7) (*p* > 0.05). In contrast, either blockade of ACE1 or activation of ACE2 in HEM rats significantly elevated plasma levels of Ang-(1-7) when compared to other groups of rats (*p* < 0.05) (one-way ANOVA for changes in Ang-(1-7) levels, *F*(5, 30) = 12.30, *p* < 0.0001) (Figure 4(b)).

### 3.5. Changes in HEM-Induced Expression of Brain RAS, PIC, and ER Stress Marker and the Effect of Cap or DIZE

**Figure 5:**
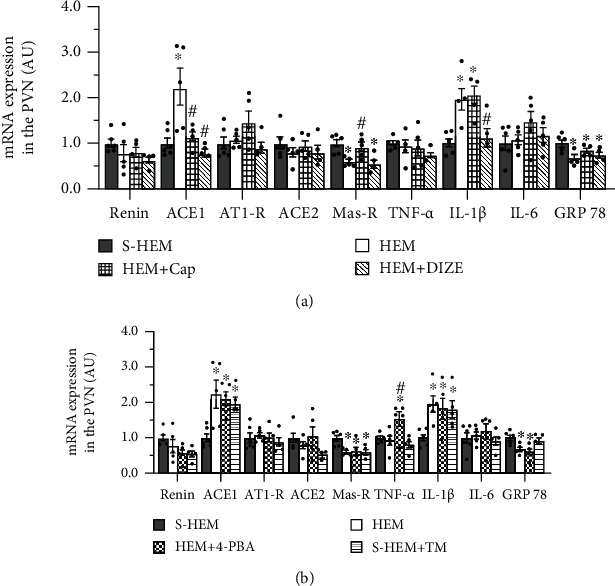
Quantitative comparison of the mRNA expression of renin-angiotensin system components, proinflammatory cytokines, and endoplasmic reticulum stress marker in the hypothalamic paraventricular nucleus (PVN) in sham hemorrhage (S-HEM), HEM, and HEM plus treatment with either captopril (Cap) or diminazene aceturate (DIZE) rats (a) or HEM plus treatment with either 4-phenylbutyric acid (4-PBA) or tunicamycin (TM) rats (b) before angiotensin II infusion (^∗^*p* < 0.05 vs. S-HEM rats; ^#^*p* < 0.05 vs. HEM rats).

In PVN tissues collected 8 days after the last HEM challenge, HEM rats exhibited upregulation of mRNA expression of the prohypertensive RAS component ACE1 and the PIC IL-1*β* and downregulation of expression of the Mas-R, an antihypertensive RAS component (*p* < 0.05, Figure 5(a)). Cap treatment reversed the increased mRNA expression of ACE1, decreased Mas-R mRNA expression (*p* < 0.05, Figure 5(a)), and maintained IL-1*β* mRNA expression high. The ACE2 agonist DIZE blocked the increased mRNA expression of ACE1 and IL-1*β* (*p* < 0.05, Figure 5(a)) but had no effect on the decreased mRNA expression of the Mas-R (one-way ANOVA for mRNA expression changes in ACE1, *F*(3, 17) = 8.794, *p* < 0.001, and Mas-R, *F*(3, 17) = 8.440, *p* < 0.0012).

Interestingly, HEM downregulated the mRNA expression of the ERS marker GRP78 in the PVN (*p* < 0.05). Treatment with either Cap or DIZE did not alter the HEM-induced effect on GRP78 mRNA expression the PVN (Figure 5(a)).

**Figure 6:**
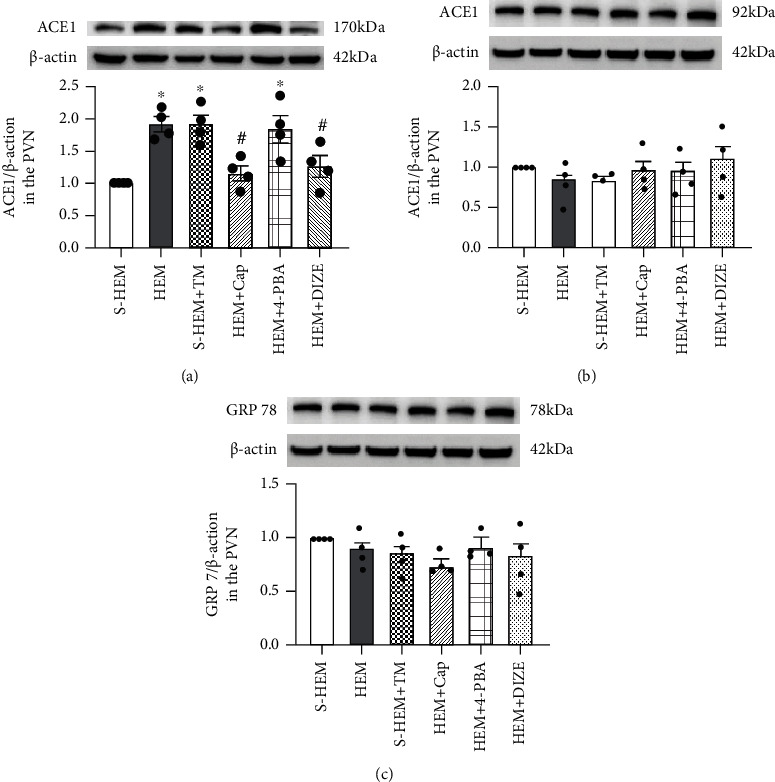
Western blot analysis of angiotensin-converting enzyme 1 (ACE1 (a)), ACE2 (b), or glucose-regulated protein 78 (GRP 78 (c)) protein expression in the hypothalamic paraventricular nucleus (PVN) in sham hemorrhage (S-HEM), HEM, and HEM plus treatment with captopril (Cap), diminazene aceturate (DIZE), 4-phenylbutyric acid (4-PBA), or tunicamycin (TM) before angiotensin II infusion. Representative Western blots of ACE1, ACE2, GRP 78, and *β*-actin and analyzed results showed the change in ACE1, ACE2, or GRP 78 protein expression, which was normalized with *β*-actin in the PVN (^∗^*p* < 0.05 vs. S-HEM rats; ^#^*p* < 0.05 vs. HEM rats).

Western blot analysis to confirm the effects of HEM on genomic expression revealed increased ACE1 protein and unaltered ACE2 and GRP 78 protein in the PVN of the HEM rats. Treatment with either Cap or DIZE significantly attenuated the increased protein expression of ACE1 protein (*p* < 0.05) but had no effects on ACE2 and GRP78 protein expression (*p* > 0.05, Figures 6(a)–6(c)) (one-way ANOVA for protein expression changes in ACE1, *F*(5, 18) = 8.714, *p* < 0.0002; ACE2, *F*(5, 18) = 1.175, *p* = 0.3598; and GPR 78, *F*(5, 18) = 1.031, *p* = 0.4292).

### 3.6. The Role of ERS in HEM-Induced Changes in Brain RAS, PICs, and ER Stress Marker

Treatment with ERS agonist TM in the S-HEM rats resulted in increased mRNA expression of ACE1 and IL-1*β* and decreased mRNA expression of Mas-R in the PVN (*p* < 0.05). Treatment with the ERS antagonist 4-PBA had no effects on the HEM-induced increase in mRNA expression of ACE1 and decrease in mRNA expression of Mas-R and GRP78, even unregulated TNF-*α* expression in the PVN (one-way ANOVA for mRNA expression changes in ACE1, *F*(3, 17) = 5.913, *p* < 0.0059, and Mas-R, *F*(3, 17) = 5.942, *p* < 0.0058) (Figure 5(b)).

Western blot analysis revealed that activation of ERS resulted in increased ACE1 protein expression in the PVN of the S-HEM rats (*p* < 0.05). However, blockade of ERS did not alter increased ACE1 protein expression in the PVN of the HEM rats (*p* > 0.05). Both activation and blockade of ERS had no effects on protein expression of ACE2 and GRP 78 in the PVN (*p* > 0.05, Figures 6(a)–6(c)) (one-way ANOVA for protein expression changes in ACE1, *F*(5, 18) = 8.714, *p* < 0.0002; ACE2, *F*(5, 18) = 1.175, *p* = 0.3598; and GPR 78, *F*(5, 18) = 1.031, *p* = 0.4292).

## 4. Discussion

The major findings of the present study are as follows: (1) in comparison to those receiving S-HEM, controlled HEM sensitized Ang II-elicited hypertension. Activation of ERS in S-HEM rats also sensitized Ang II-elicited hypertension. These sensitized hypertensive responses were mediated by increased sympathetic tone; (2) either systemic blockade of ACE1 or activation of ACE2 abolished the HEM-induced elevation of sympathetic outflow and sensitization of hypertension. However, blockade of ERS in HEM rats did not alter these HEM-induced effects; (3) either HEM or activation of ERS elevated plasma levels of Ang II but did not alter plasma levels of Ang-(1-7). Blockade of ACE1 or activation of ACE2, but not inhibition of ERS, reduced the increase in Ang II and elevated Ang-(1-7) plasma levels; (4) in the PVN, mRNA and protein expression of a RAS prohypertensive component, ACE1, was significantly elevated by HEM and activation of ERS, but mRNA and protein expression of a RAS antihypertensive component, ACE2, was not evident. However, these treatments downregulated mRNA expression of the Ang-(1-7) receptor Mas-R in the PVN. Blockade of ACE1 or activation of ACE2, but not ERS blockade, reversed HEM-induced changes in expression of RAS components; and (5) although some treatments changed PVN mRNA expression of the ERS marker GRP 78, the protein expression of GRP 78 was not altered. Taken together, the results of this series of experiments indicate that controlled HEM-induced increased RAS prohypertensive activity and decreased RAS antihypertensive activity resulting in augmented central sympathetic drive, thereby accounting for the actions of the brain RAS in sensitization of the Ang II hypertensive response. Under the HEM conditions applied in the current studies, ERS apparently is not involved in mediating HTRS induced by hypotensive hemorrhage. The present study provides evidence for hemorrhage as a novel risk factor that increases the vulnerability to hypertension in individuals after severe blood loss.

The role of the RAS and AVP as humoral components in maintaining BP during hemorrhage is well established. The release of AVP into the circulation and the activation of brain RAS increase central drive of the SNS elevating renal and splanchnic sympathetic nerve activity (SNA), all of which play important counter regulatory roles to reestablish cardiovascular homeostasis and stability during and after hemorrhage [6–10]. In this regard, information indicative of increased activity of the systemic RAS and AVP is communicated to the CNS by both humoral and neural afferent signaling [41]. The PVN receives information about blood-borne or extracellular humoral factors (e.g., Ang II; [Na^+^]) through the SFO and OVLT (regions lacking a blood-brain barrier). This humoral information is integrated with neural input that is initially derived from systemic volume and BP receptors [42, 43]. Efferent from the PVN projects to the RVLM and spinal cord, regions that control sympathetic tone and BP [44]. Therefore, it is likely that recovery from hypotensive hemorrhage by sympathetic and RAS activation and vasopressin release is via the peripheral to central, humoral-neural coupling pathway and the integrity of the PVN [7, 15, 16, 19].

Do and colleagues observed markedly increased angiotensinogen mRNA levels in the rat hypothalamus and brainstem after hemorrhage [45]. Central blockade of AT1-R produced a markedly greater fall in BP, a reduced tachycardia, and impaired vasopressin release during and after hemorrhage [7, 11], suggesting that the brain RAS is stimulated by hemorrhage. In addition, many studies have provided compelling evidence that hemorrhage can trigger excessive PIC release in the periphery and CNS [12, 35]. The RAS and inflammatory response reciprocally activate each other in various pathophysiological conditions [22]. Although the activation of the RAS and inflammation following hemorrhage play an important beneficial role in the short-term by compensating for hypovolemic hypotension, these responses to restore homeostasis have long-term cardiovascular consequences. In the present study, we found that controlled hemorrhage significantly induced sustained increases in plasma levels of Ang II and mRNA and protein expression of ACE1 and the PIC IL-1*β* in the PVN. These changes might reflect a mechanism for sensitizing the brain cardiovascular nuclei and enhance their reactivity, which produces increased centrally driven sympathetic activity. As a result, Ang II infusion elicited an augmented hypertensive response in rats subjected to hemorrhage earlier in life. In contrast, systemic blockade of ACE1 reversed the increased plasma Ang II levels and expression of prohypertensive component and decreased expression of an antihypertensive component (Mas-R) produced by hemorrhage resulting in a significant reduction of centrally driven sympathetic activity and attenuation of the hemorrhage-induced sensitizing effect on hypertension. These results suggest that the hemorrhage-induced upregulation of components of the RAS and PIC in the CNS participates in the sensitization process that predisposes the individuals suffering hemorrhage to the expression of frank hypertension later in their lifetime. This is consistent with previous studies from Xue and colleagues showing that a wide range of physiological or psychological stressors upregulate central RAS and inflammation to mediate hypertensive response sensitization, which was blocked by blockade of ACE1 [21, 22, 46].

Besides the prohypertensive arm of the RAS (ACE1/Ang II/AT1-R), an antihypertensive arm of the RAS has been identified in the brain and in the periphery. The antihypertensive arm includes ACE2, Ang-(1-7), and the Mas-R [26]. ACE2 is present in many brain regions including the PVN [47]. Numerous studies have shown that ACE2 has functional properties that oppose the actions of the classic RAS by favorably regulating cardiovascular function, BP, and baroreflex sensitivity through production of Ang (1-7) [26]. In the brain, Ang-(1-7) is also colocalized with nitric oxide synthase (NOS) in the PVN to stimulate NO production through activation of the Mas-R [48, 49]. It has been shown that PVN NO can tonically inhibit SNA and AVP release [50]. Whitaker and Molina reported that PVN NOS activity and NO were significantly higher in acute ethanol-intoxicated rats following hemorrhage. Central blockade of Mas-R decreased NOS activity and NO concentration, partially restoring the increase in circulating AVP level at completion of hemorrhage in acute ethanol-intoxicated rats. This suggests that activation of ACE2/Ang-(1-7)/Mas-R pathway contributes to tonic inhibition of vasopressin release during hemorrhagic shock in rodents [17, 18]. Based on these results, one can speculate that hemorrhage may downregulate the antihypertensive arm of the RAS to reduce NO production, which would reduce the inhibitory effect of NO on the SNA and AVP release that antagonize hypotension produced by hemorrhage. Consistent with this notion, we found that hemorrhage downregulated the mRNA expression of the Ang-(1-7) receptor Mas-R in the PVN. However, this downregulated antihypertensive arm of the RAS is likely to attenuate the antagonism for the sensitizing effect of the RAS prohypertensive arm, which may also play an augmented role in the sensitization of Ang II-elicited hypertension. Previous studies demonstrated that central administration of Ang-(1-7) downregulated the mRNA expression of RAS hypertensive components in the LT and blocked Ang II-induced sensitization of hypertension in male rats [51]. In the present study, we found that administration of an ACE2 activator inhibited hemorrhage-elicited increases in plasma Ang II levels, increased plasma levels of Ang-(1-7), reversed the increased expression of RAS prohypertensive component and a PIC, and attenuated the reduction of BP in response to ganglionic blockade. All these effects would tend to contribute to the attenuation of hemorrhage-induced sensitization of hypertension. Our present results confirm and extend the point that activation of antihypertensive arm of the RAS plays a protective role in the induction of HTRS after hemorrhage.

The ER is the cellular organelle responsible for synthesis, maturation, and trafficking of a wide range of proteins. Disruption of ER function can cause an accumulation of misfolded or unfolded proteins in the cell and create a condition known as ERS [52, 53]. Three classes of ERS transducers, including inositol-requiring kinase 1, protein kinase RNA-like ER kinase–translation initiation factor *α* (eIF2*α*), and transcriptional factor activating transcription factor-6, have been identified. All three sensors are maintained in an inactive state through an interaction with GRP78, the ERS chaperone. When unfolded proteins accumulate in the ER, GRP78 is rapidly dissociated from the ER sensors to initiate the unfolded protein response [54]. Studies have demonstrated that activation of brain ERS mediates Ang II infusion-induced hypertension and BP increase in the SHR [31, 32]. Both hypertensive animal models showed that GRP78 and other ERS markers were increased in the SFO and RVLM and that inhibition of ERS in these brain regions prevented these forms of hypertension [31, 32]. Recently, emerging evidence showed that hemorrhage is associated with activation of ERS, which has been suggested to be a result of overactivation of the RAS and elevated inflammation following hemorrhage [12, 13]. These studies may also suggest that ERS is involved in sensitization of hypertension induced by hemorrhage. However, in the present study, we found that blockade of ERS in HEM animals did not attenuate the HEM-induced sensitization of hypertension. Also, hemorrhage had no effect on protein expression of ERS chaperone GRP 78 in the PVN and blockade of ERS did not alter HEM-induced gene and protein expression of ACE1 and GRP 78 in the PVN. These results indicate that ERS under the experimental conditions employed in the present studies may not mediate HEM-induced HTRS. Under more extreme conditions [12, 35, 55], perhaps hemorrhage would initiate changes in ERS that are sustained and mediate the sensitized hypertensive response. Further studies on this issue are warranted.

In the present study, we also found that like the sensitizing effect of hemorrhage, activation of ERS in S-HEM rats resulted in sensitization of Ang II-elicited hypertension and that this was accompanied by increased expression of RAS prohypertensive and inflammatory indicators, but not of the ERS marker GRP 78. Previous studies have demonstrated that activation of ERS in the SFO and RVLM is responsible for the increased BP in both SHR and Ang II infused rats. Inhibition of ERS in these brain regions prevented hypertension in these models [31, 32]. Therefore, it is possible that ERS activation occurred in other cardiovascular nuclei such as the SFO and RVLM rather than in the PVN.

A limitation of the current study in examining the RAS antihypertensive arm was that although we determined both mRNA and protein expressions of ACE2, we only determined message for the Mas-R. Protein expression of the Mas-R is problematic since reliable specific antibodies for the Mas-R are not readily available [56]. This requires the development of new tools to confirm the antagonizing effect of RAS antihypertensive arm on sensitization of hypertension induced by various challenges including hemorrhage.

## 5. Conclusion and Perspectives

**Figure 7:**
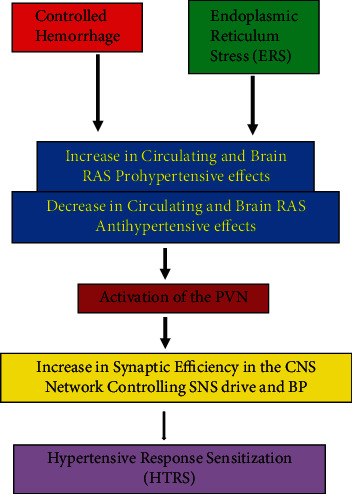
Schematic representation of controlled hemorrhage-induced hypertensive response sensitization (HTRS) through upregulation of renin-angiotensin system (RAS) prohypertensive effects and downregulation of RAS antihypertensive effects in the peripheral and central nervous system (CNS) network controlling sympathetic nervous system (SNS) drive and blood pressure (BP) including the paraventricular nucleus of hypothalamus (PVN). Endoplasmic reticulum stress (ERS) does not appear to mediate the induction and maintenance of a sensitized state after hypotensive hemorrhage although activation of ERS results in sensitization of angiotensin II-elicited hypertension in the present study.

This study demonstrated that controlled hemorrhage induces HTRS probably through shifting the balance of the prohypertensive and antihypertensive pathways in favor of enhanced central activity driving SNA and elevated BP (Figure 7). Blockade of the RAS prohypertensive arm or activation of the RAS antihypertensive arm leads to attenuated overall activity of the RAS and reduced inflammation that is effective in lowering centrally driven sympathetic activity and blocking the induction of HTRS. This study provides insights into central mechanisms that increase the predisposition for development of hypertension after hemorrhage.

Hemorrhage accounts for 30-40% of total trauma deaths. Blood transfusion with balanced components or whole blood for patients suffering from HS has dramatically increased survival [2–4]. However, the long-term consequences of HS and the development of chronic disorders, such as cardiovascular diseases including hypertension, and interventions to reduce such pathologies in survivors of HS have not been reported. The present study provides the first direct evidence implicating the sensitizing effects of hemorrhage on the development of hypertension and reveals the central mechanisms of the RAS and sympathetic activation in the sensitization process. Manipulation of the RAS by inhibition of its prohypertensive effects and activation of its antihypertensive effects antagonizes hemorrhage-elicited sensitization of hypertension, highlighting a potential target for pharmacologic therapy and management strategies of long-term cardiovascular consequences related to hemorrhage.

## Figures and Tables

**Table 1:** Primer sequences for real-time PCR.

Gene	Forward primer	Reverse primer	Product size (bp)
GAPDH	TGACTCTACCCACGGCAAGTTCAA	ACGACATACTCAGCACCAGCATCA	141
Renin	CTGCCACCTTGTTGTGTGAG	ACCTGGCTACAGTTCACAACG	154
ACE1	GTGTTGTGGAACGAATACGC	CCTTCTTTATGATCCGCTTGA	187
AT1-R	CTCAAGCCTGTCTACGAAAATGAG	GTGAATGGTCCTTTGGTCGT	188
ACE2	TTAAGCCACCTTACGAGCCTC	GCCAATGTCCATGGAGTCAT	170
Mas-R	TGTGGGTGGCTTTCGATT	CCCGTCACATATGGAAGCAT	159
TNF-*α*	GCCGATTTGCCACTTCATAC	AAGTAGACCTGCCCGGACTC	209
IL-6	GCCTATTGAAAATCTGCTCTGG	GGAAGTTGGGGTAGGAAGGA	160
IL-1*β*	AGCAACGACAAAATCCCT GT	GAAGACAAACCGCTTTTCCA	209
GRP 78	TTCCGAGGAACACTGTGGTG	GTCAGGGGTCGTTCACCTTC	109

ACE: angiotensin-converting enzyme; AT1-R: angiotensin II type 1 receptor; Mas-R: angiotensin-(1–7) receptor; TNF-*α*: tumor necrosis factor-*α*; IL-1*β*: interleukin-1*β*; IL-6: interleukin-6; GRP 78: the glucose-regulated protein 78.

## Data Availability

All supporting data for this manuscript are included in the figures and available from the corresponding authors on reasonable request.

## Abbreviations

ACE: Angiotensin-converting enzyme
Ang II: Angiotensin II
AT1-R: Angiotensin II type 1 receptor
AVP: Arginine vasopressin
BP: Blood pressure
Cap: Captopril
CNS: Central nervous system
DIZE: Diminazene aceturate
ERS: Endoplasmic reticulum stress
GRP 78: Glucose-regulated protein 78
HEM: Hemorrhaged
Hex: Hexamethonium
HR: Heart rate
HS: Hemorrhagic shock
HTRS: Hypertensive response sensitization
IL-1*β*: Interleukin-1*β*
IL-6: Interleukin-6
LT: The lamina terminalis
MAP: Mean arterial pressure
MnPO: Median preoptic nucleus
OVLT: The vascular organ of the lamina terminalis
4-PBA: 4-Phenylbutyric acid
PIC: Proinflammatory cytokine
PVN: Hypothalamic paraventricular nucleus
RAS: Renin-angiotensin system
RVLM: Rostral ventrolateral medulla
SFO: The subfornical organ
S-HEM: Sham hemorrhaged
SHRs: Spontaneously hypertensive rats
SNA: Sympathetic nerve activity
SNS: Sympathetic nervous system
TNF-*α*: Tumor necrosis factor-*α*
TM: Tunicamycin.
