# Correction to “Controlled Hemorrhage Sensitizes Angiotensin II‐Elicited Hypertension Through Activation of the Brain Renin‐Angiotensin System Independently of Endoplasmic Reticulum Stress”

**DOI:** 10.1155/omcl/9829207

**Published:** 2026-01-07

G.‐B. Wu, H.‐B. Du, J.‐Y. Zhai, S. Sun, J.‐L. Cui, Y. Zhang, Z.‐A. Zhao, J.‐L. Wu, A. K. Johnson, B. Xue, Z.‐G. Zhao, and G.‐S. Zhang, “Controlled Hemorrhage Sensitizes Angiotensin II‐Elicited Hypertension Through Activation of the Brain Renin‐Angiotensin System Independently of Endoplasmic Reticulum Stress,” *Oxidative Medicine and Cellular Longevity* 2022, no. 1 (2022): 6371048, https://doi.org/10.1155/2022/6371048.

In the article, there are errors in Figure 6. Specifically:

• The ACE1 western blots in Figure 6b were incorrectly labeled and should represent ACE2.
• The western blots presented in Figure 6c were erroneously duplicated from the blots shown in Figure 6b.

Both errors were introduced during the production process. The correct Figure 6 is shown below:

Figure 6Western blot analysis of angiotensin‐converting enzyme 1 (ACE1 (a)), ACE2 (b), or glucose‐regulated protein 78 (GRP 78 (c)) protein expression in the hypothalamic paraventricular nucleus (PVN) in sham hemorrhage (S‐HEM), HEM, and HEM plus treatment with captopril (Cap), diminazene aceturate (DIZE), 4‐phenylbutyric acid (4‐PBA), or tunicamycin (TM) before angiotensin II infusion. Representative Western blots of ACE1, ACE2, GRP 78, and β‐actin and analyzed results showed the change in ACE1, ACE2, or GRP 78 protein expression, which was normalized with β‐actin in the PVN ( ^∗^ *p* < 0.05 vs. S‐HEM rats;  ^*#*^ *p* < 0.05 vs. HEM rats).

**Figure:**
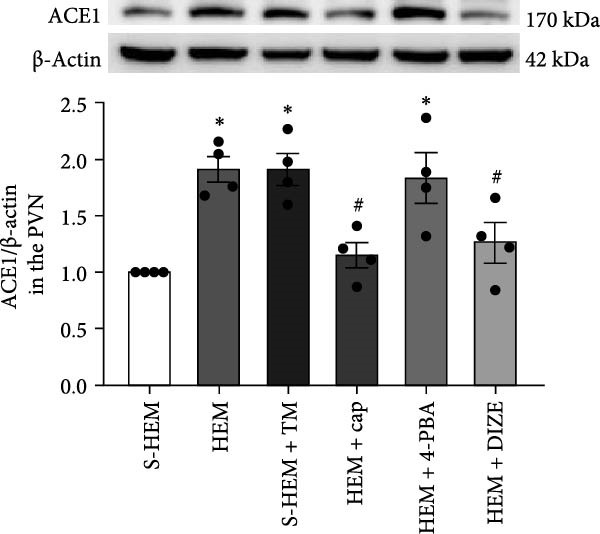
(a)

**Figure:**
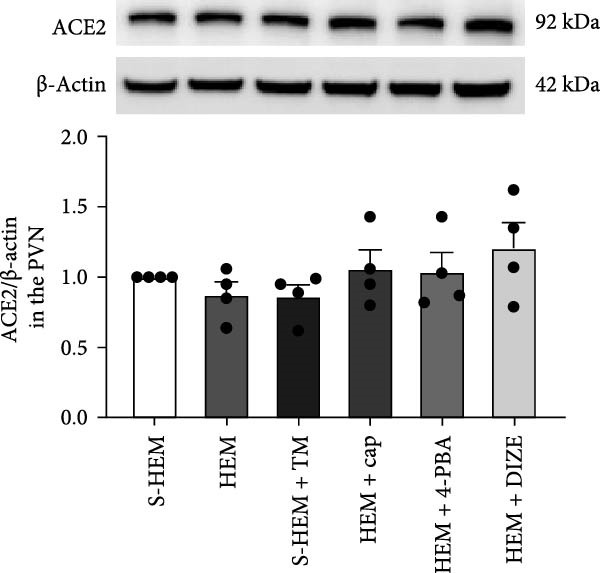
(b)

**Figure:**
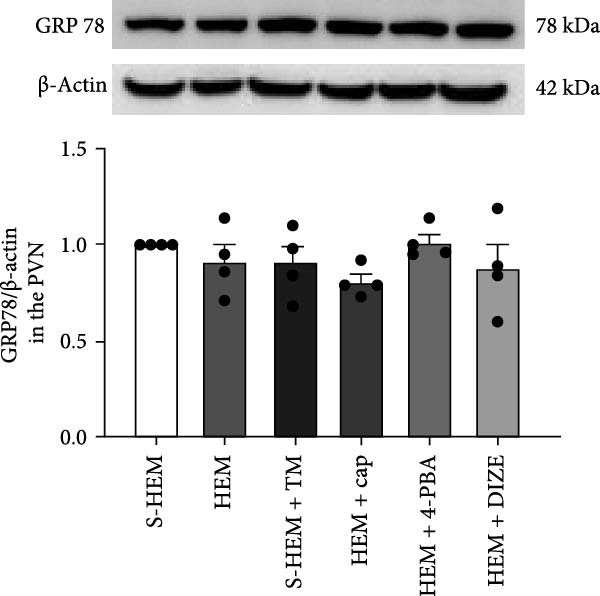
(c)

We apologize for these errors.

